# Hip Tenosynovial Giant Cell Tumor without Recurrence in a 9-Year Follow-Up

**DOI:** 10.1155/2022/1797218

**Published:** 2022-02-04

**Authors:** Seyyed-Morteza Kazemi, Seyyed-Mohsen Hosseininejad

**Affiliations:** ^1^Bone Joint and Related Tissues Research Center, Akhtar Orthopedic Hospital, Shahid Beheshti University of Medical Sciences, Tehran, Iran; ^2^Clinical Research & Development Unit, Akhtar Hospital, Shahid Beheshti University of Medical Sciences, Tehran, Iran; ^3^Joint, Bone, Connective Tissue Rheumatology Research Center (JBCRC), Golestan University of Medical Sciences, Gorgan, Iran

## Abstract

**Background:**

Tenosynovial giant cell tumors are a benign but rare condition with potentially aggressive tumor-like traits which should be considered in young patients with monoarticular joint involvement. *Case Presentation*. This report presents a 31-year-old otherwise healthy woman with a right hip pain. Clinical and histopathological investigations revealed the diagnosis of diffuse-type tenosynovial giant cell tumor of the hip (the diffuse intra-articular form of PVNS). Open synovectomy and tumor resection and surgical dislocation of the hip were performed. She was free of symptoms and recurrence within a 9-year-follow-up period.

**Conclusion:**

Open synovectomy and tumor resection through surgical dislocation of the hip without adjutant radiotherapy could be a reliable choice for the localization of the hip.

## 1. Introduction

Tenosynovial giant cell tumor (TGCT) is a locally aggressive mesenchymal entity previously known as pigmented villonodular synovitis (PVNS) which is a rare condition affecting small and large joints' synovium, tendon sheaths, and bursa of unknown etiology with a yearly incidence of 1.8 per million, generally seen in the third to fifth decade of life [1–3] with a slight preponderance for females [4–8]. TGGC could have proliferative and tumor-like behavior resulting in unswerving joint degradation through bone and cartilage penetration in spite of being introduced as a benign process [9]. This condition clinically presents with pain, swelling, and a restricted range of motion of the affected joint [6, 10]. The duration of symptoms could be one to 120 months with an average of 15 months with chronic intermittent symptoms in most cases [7]. Given the nonspecific presentations, the diagnosis of TGCT is often delayed; radiographs might be remarkable in up to 21% of cases [11, 12]. The diagnosis is generally made on magnetic resonance imaging [13]. It typically appears as intra-articular focal nodules and effusion of low signal on both T1- and T2-weighted images due to hemosiderin deposition, heterogeneous thick fibrous tissue, synovial hyperplasia, and bone erosion with conserved bone density and joint space width which can also be detected on plain X-rays as well [1, 14, 15].

The primary treatment option is open total tumor resection and synovectomy to preserve joint function, which rarely needs revision surgery with an average time to revision of 6.5 years in previous cases [3, 16, 17]. There might be 8–56% chance of recurrence which might entail adjuvant radiation therapy in some cases [18–20]. TGCT of the hip joint is a comparatively rare condition, as the hip is affected in 15% of total cases [16, 21]. Here, we aim to introduce a case of the hip TGCT without recurrence postoperatively in a 9-year follow-up.

## 2. Case Presentation

A 31-year-old otherwise healthy civil engineer female referred to our orthopedic clinic with a complaint of an atraumatic right hip pain, inability to bear weight, and a limited range of motion for one month following a long walking journey outdoors in nature. The pain was dominant at night, exacerbating with pressure or movement. The patient past medical history was negative for any condition except taking Rakuten tablets for acne in several periods. She had received conservative treatments during the recent one month, and even a rheumatologic and infectious condition had been ruled out but no improvement of symptoms occurred.

On the physical examination, the hip range of motion in all directions was limited beside a local tenderness in the hip but without swelling or erythema.

**Figure 1:**
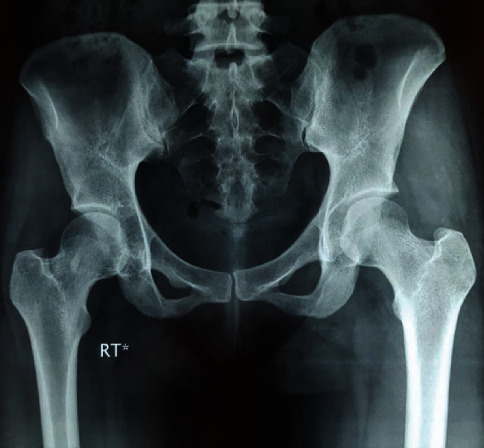
Preoperative anteroposterior pelvic X-ray of a 31-year-old female shows narrowing of joint space and cortical erosions and lucency in the acetabulum and femoral head of the right hip.

She had received conservative treatments but no improvement of symptoms occurred. Laboratory blood tests were normal. Initial pelvic radiographs showed joint space involvement and cortical erosion in the right acetabulum and femoral head (Figure 1).

**Figure 2:**
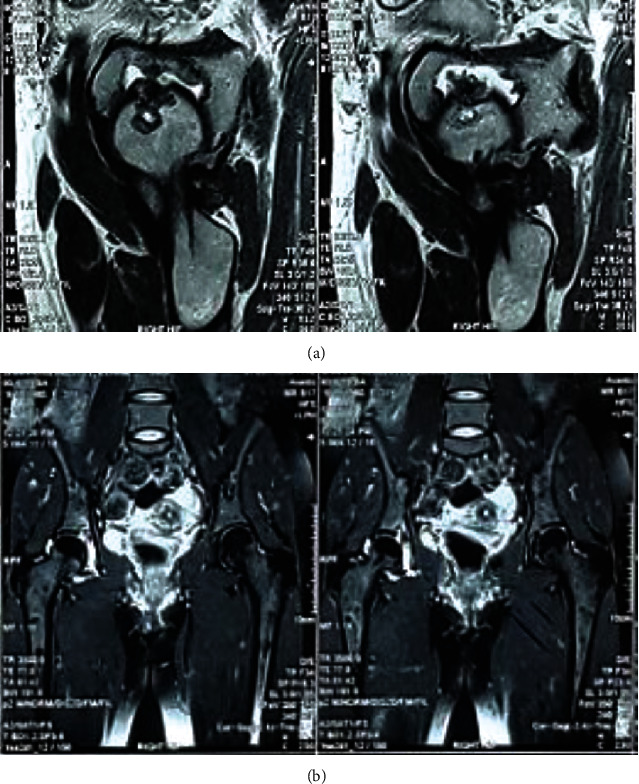
Synovium appears as hyposignal on T2-wieghted preoperative MRI in the sagittal plane and enhances after the gadolinium injection (a). Preoperative MRI showing the characteristic synovial process with low-signal intensity in fat-suppressed T2-weighted sequences (b).

MRI of the hip showed characteristic low-signal intensity synovial involvement because of high hemosiderin content (Figure 2). On Tc-99m-methylene diphosphonate, whole-body bone scintigraphy noted right hip joint hyperemia, suggestive for inflammatory condition.

**Figure 3:**
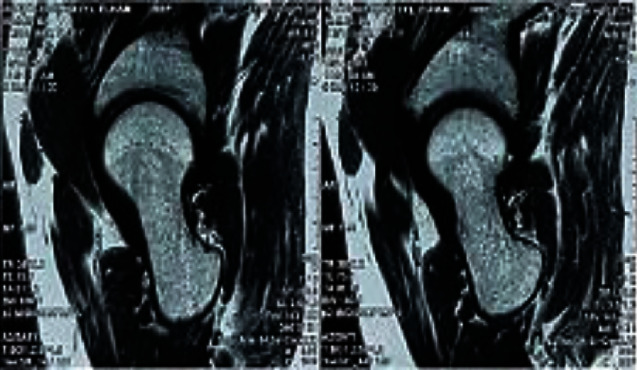
T2-weighted MRI of the hip showing normal condition of the hip cleared from any abnormal lesion postoperatively.

**Figure 4:**
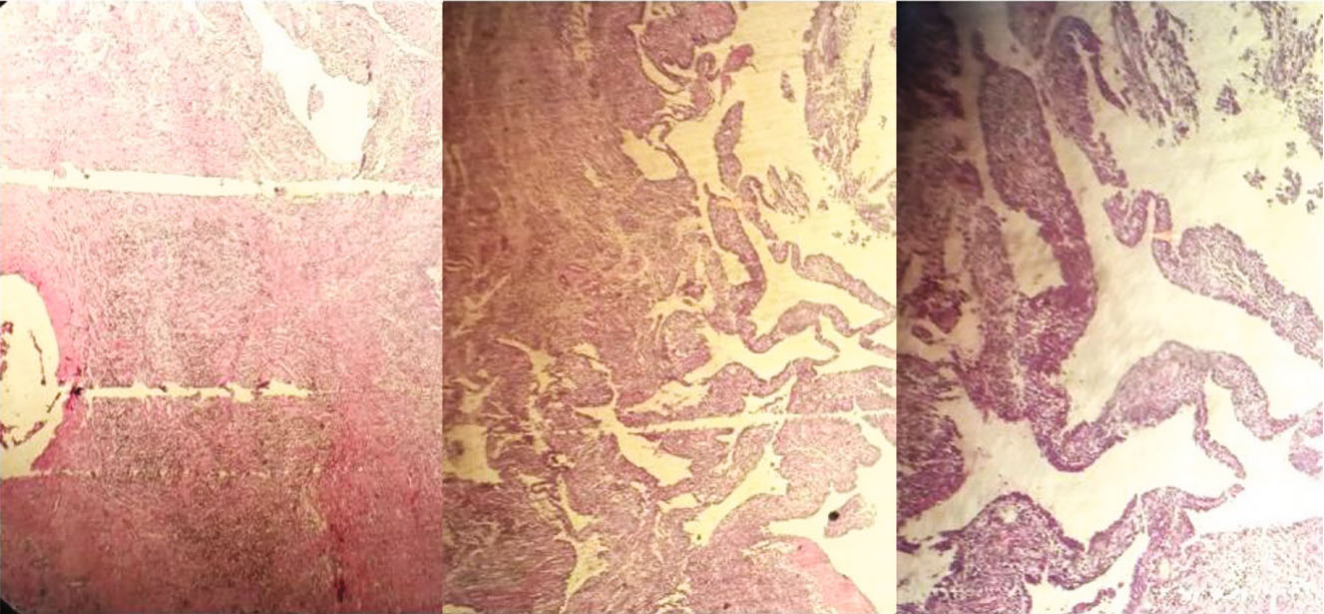
Histopathology appearance of TGCT shows diffuse-type intra-articular lesion, which indicates hemosiderin deposition and villonodular protrusions with overlying synovial tissue; mononuclear histiocytoid cells which interacted with osteoclast-like multinucleated giant cells are also noted.

The diagnosis of diffuse-type giant cell tumor was made and the open surgical synovectomy was planned. We conducted surgical dislocation of the joint using the technique described by Ganz et al. [22] under spinal anesthesia; the joint's entire synovium and the synovium around ligamentum teres (LT) were involved, without involvement of the femoral neck retinaculum. So, total tumor resection and synovectomy of the joint including LT and its femoral and acetabular attachment were performed besides debridement of the juxta-articular subchondral bone; postoperative MRI findings showed the absence of the synovial lesion (Figure 3). Postoperative synovial biopsy confirmed the diagnosis of TGCT as well (Figure 4).

**Figure 5:**
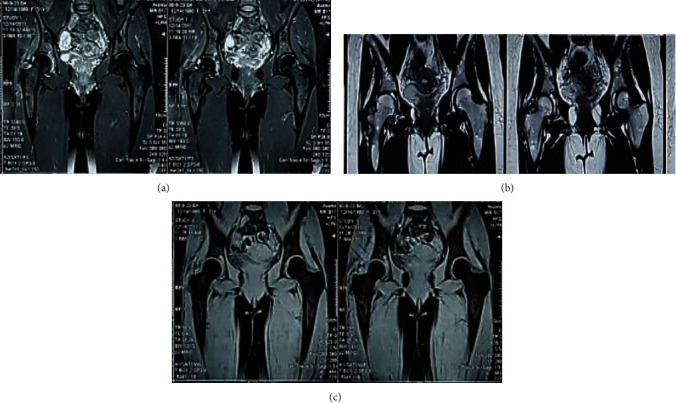
Proton density-weighted (a) MRI of the hip showing normal condition of the hip cleared from any abnormal lesion in a 6-year-follow-up visit. Postoperative 9-year follow-up T2 (b) and fat-suppressed (c) coronal MRI of the hip showing no lesion recurrence in the right hip synovial involvement.

**Figure 6:**
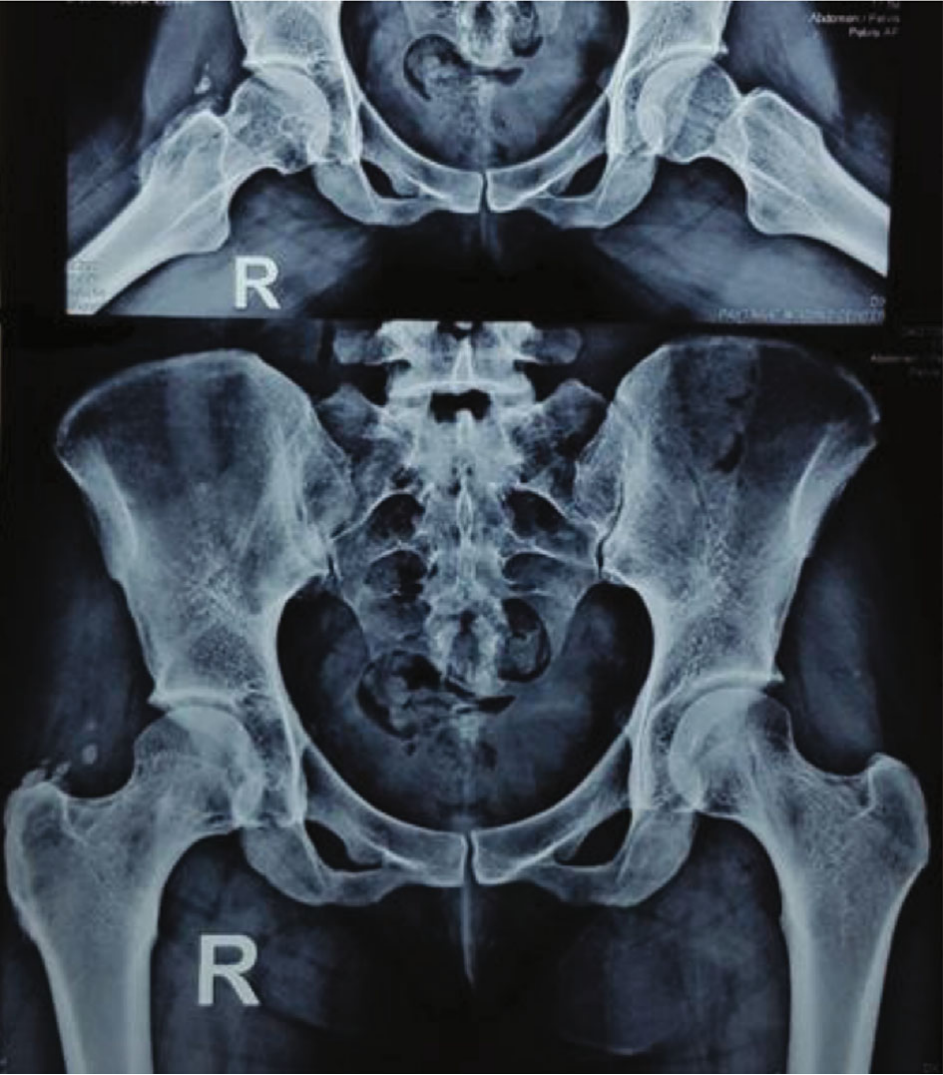
Latest postoperative follow-up X-ray supporting disease-free condition of the right hip.

The patient was discharged the next day after surgery with good general condition. She was ordered to come for follow-up visit after 3 and 6 months and then annually with control MRI which all showed no recurrence of the lesion (Figure 5); a latest hip X-ray also supported the disease-free condition (Figure 6). The patient is now 40 years old and fell symptom free during the last 9 years and does her job normally and has even continued going journey outdoors in nature with no serious complaint.

## 3. Discussion

TGCT is an infrequent, benign but proliferative disease of the synovium of especially large joints, occurring about 15% in the hip joint. Though an inflammatory or neoplastic origin has been proposed, the exact etiology is still under debate. It is categorized as a localized form or nodular tenosynovitis, diffuse-type giant cell tumor, and PVNS. The diffuse-type tenosynovial giant cell tumor could be either purely extra-articular, located in the soft tissues, or representing the extra-articular component of a PVNS; the histopathologic picture of these three lesions is rather alike consisting of elongated synovial villi and synoviocyte line besides sheets of mononuclear cells intermingled with osteoclast-like multinucleated giant and siderophage and xanthoma cells [16, 23]; hemorrhage and inflammatory infiltration can occur in the lesion [24]. The hip TGCT is frequently of diffuse-type PVNS [25]. Pain is the main symptom which increases in duration and intensity with the progression of the lesion. Pain is often localized in the groin but may be felt throughout the entire hip area. Other clinical signs include chronic intra- or extra-articular painful swelling associated with the joint's limitation of range of motion and joint stiffness (7). The functional impairments emerge later while the intracapsular joint space decreases subsequent to the synovial invasion. Patients' tolerance to pain, its gradual development, the varied symptoms, the difficult palpation of the hip joint, and the normal appearance of the early radiographs of the condition yield in a nonspecific presentation, which frequently delay making the diagnosis, taking averagely 18 to 48 months [1, 2, 10, 12, 16, 26].

While the nonappearance of juxta-articular osteophytes and osteopenia is typical in TGCT [24], dense soft tissue swelling, scalloping bony erosions with sclerotic borders, and joint space preservation in the early stage of the condition are the key features of TGCT seen on plain radiographs [24]. However, MRI would serve as the most proper imaging study to detect and assess the extension of disease in TGCT [11].

For diffuse-type TGCT of the hip, early entire tumor resection and synovium are needed to limit articular destruction and the potential recurrence. The primary treatment options include tumor resection and synovectomy alone and synovectomy plus arthroplasty, which consists of total hip arthroplasty, hemiarthroplasty, cup arthroplasty, and hip resurfacing. Even if total synovectomy seems to be effective to prevent recurrence, according to Vastel et al., it does not postpone the development of secondary osteoarthritis of the hip following synovial disturbance, necessitating a total hip replacement procedure [5, 16].

Excision is sufficient for local PVNS through arthroscopy or arthrotomy; however, if the lesion is diffuse, total synovectomy is compulsory [1, 3, 14, 17, 27, 28]. Tumor resection and open synovectomy serve as the most broadly labeled treatment options for addressing hip TGCT [16]. As stated in the literature, tumor resection and synovectomy-only approach could induce a higher recurrence rate; the least average time to revision surgery is 6.5 years [17]. Applying adjuvant radiotherapy for PVNS is under debate [1, 29]; synovectomy and added external radiotherapy showed some functional improvement and improvement in patients' quality of lives with a low rate of lesion recurrence in the knee [30]. However, efficacy is unconfirmed and there is caution against unwelcome irradiation effects around the reproductive organs, e.g., the hip and pelvis [6, 31, 32].

In our case, open synovectomy with a Ganz surgical dislocation of the hip was performed because it was essential to excise total synovium and for clearing of the acetabular fossa. As no recurrence occurred in the 9-year follow-up, we had no need for surgical revision. Adjuvant therapy was not conducted as well, as it was not necessary and to avoid unwanted adverse effects on adjacent reproductive organs.

## 4. Conclusion

A provocative onset of hip pain could be a clinical presentation for diffuse-type TGCT, so physicians should consider such condition.

Open synovectomy and resection of tumor through surgical hip dislocation without adjuvant radiotherapy could serve as reliable techniques to clear the joint space from pathologic synovium, with no chance of recurrence, as reported in the current case. However, further trail studies seem to be conducted to investigate the outcome of this kind of treatment approach in the condition.

## Data Availability

The data of the case has been given in the manuscript body.
